# An Analysis of the Myocardial Transcriptome in a Mouse Model of Cardiac Dysfunction with Decreased Cholinergic Neurotransmission

**DOI:** 10.1371/journal.pone.0039997

**Published:** 2012-06-29

**Authors:** Ashbeel Roy, Aline Lara, Diogo Guimarães, Rita Pires, Eneas R. Gomes, David E. Carter, Marcus V. Gomez, Silvia Guatimosim, Vania F. Prado, Marco A. M. Prado, Robert Gros

**Affiliations:** 1 Robarts Research Institute, Schulich School of Medicine and Dentistry, Western University, London, Ontario, Canada; 2 Departments of Physiology and Pharmacology, Schulich School of Medicine and Dentistry, Western University, London, Ontario, Canada; 3 Departments of Anatomy and Cell Biology, Schulich School of Medicine and Dentistry, Western University, London, Ontario, Canada; 4 Department of Medicine (Clinical Pharmacology), Schulich School of Medicine and Dentistry, Western University, London, Ontario, Canada; 5 Departments of Physiology and Biophysics, Institute of Biological Sciences, Federal University of Minas Gerais, Belo Horizonte, Minas Gerais, Brazil; 6 Graduate Program Santa Casa, Belo Horizonte, Minas Gerais, Brazil; University of Hong Kong, Hong Kong

## Abstract

Autonomic dysfunction is observed in many cardiovascular diseases and contributes to cardiac remodeling and heart disease. We previously reported that a decrease in the expression levels of the vesicular acetylcholine transporter (VAChT) in genetically-modified homozygous mice (VAChT KD^HOM^) leads to decreased cholinergic tone, autonomic imbalance and a phenotype resembling cardiac dysfunction. In order to further understand the molecular changes resulting from chronic long-term decrease in parasympathetic tone, we undertook a transcriptome-based, microarray-driven approach to analyze gene expression changes in ventricular tissue from VAChT KD^HOM^ mice. We demonstrate that a decrease in cholinergic tone is associated with alterations in gene expression in mutant hearts, which might contribute to increased ROS levels observed in these cardiomyocytes. In contrast, in another model of cardiac remodeling and autonomic imbalance, induced through chronic isoproterenol treatment to increase sympathetic drive, these genes did not appear to be altered in a pattern similar to that observed in VAChT KD^HOM^ hearts. These data suggest the importance of maintaining a fine balance between the two branches of the autonomic nervous system and the significance of absolute levels of cholinergic tone in proper cardiac function.

## Introduction

In many cardiovascular diseases an over-activation of sympathetic tone coupled with a decrease in parasympathetic tone is observed [1], [2], [3]. This leads to an imbalance between the two branches of the autonomic nervous system (ANS) that seem to contribute to cardiac remodeling. The ANS is the main regulator of cardiac output and, as such, plays a vital role in maintaining proper heart function. Recently, we reported that a systemic reduction in the vesicular acetylcholine transporter (VAChT; [4]), the protein responsible for packaging ACh into synaptic vesicles at parasympathetic nerve terminals, leads to a phenotype which resembles cardiac dysfunction in mice [5]. The mutant hearts have altered calcium handling and show changes in myocyte contractility, causing decreased left ventricular fractional shortening in the VAChT mutant mice [5]. Furthermore, these pathological changes can be reversed through treatment with pyridostigmine, a peripheral cholinesterase inhibitor, thus suggesting that cardiac dysfunction in these mice results from a reduction in cholinergic tone [5].

These results are in agreement with a number of recent publications which indicate that increased levels of ACh can be protective and increase survival in experimental models of heart failure. For example, vagal stimulation improves outcome in experimental models of heart failure in rats [6]. It has also been reported that chronic treatment with the cholinesterase inhibitor donepezil, an anti-Alzheimer’s drug, can reduce both cardiac hypertrophy and remodeling and increase survival rates in rat and mouse models of heart failure [7], [8]. Acetylcholine, released from vagal nerve terminals, has also been shown to have cardioprotective effects through its ability to activate the phosphatidylinositol-3-kinase (PI3K)-Akt pathway [6]. Moreover, a switch to a cholinergic phenotype occurs in sympathetic terminals in mice as well as in humans in heart failure and genetic inhibition of this transdifferentiation in mice worsens outcomes in experimental heart failure [9]. Hence, whereas higher levels of acetylcholine seem to be cardioprotective, lower levels are usually related to increased heart dysfunction.

In order to gain insight into potential molecular changes that may occur in the heart under conditions of chronically decreased cholinergic neurotransmission, we undertook a microarray-driven, transcriptome-based analysis of hearts from VAChT KD^HOM^ and wild-type (WT) mice. Our goal was to determine whether long-term decrease in ACh levels leads to alterations in gene expression profiles that could contribute to heart dysfunction.

We demonstrate here that chronic reduction in cholinergic neurotransmission in VAChT mutant mice is associated with altered gene expression in the heart. Of the changes observed, increased levels of the enzyme purine nucleoside phosphorylase could contribute to increased reactive oxygen species (ROS) levels. We found indeed that cardiomyocytes of mutant mice present an increase in ROS. Interestingly, we demonstrate that the alterations in gene expression observed in ventricles from VAChT mutant mice are distinct from those observed in a model of cardiac dysfunction with increased sympathetic drive, indicating the importance of absolute levels of cholinergic tone in regulating cardiac function.

## Methods

### Animal Models and Drug Administration

VAChT KD^HOM^ mice were generated as previously described [4]. Three-month old male wild-type (WT) and VAChT mutant mice in a mixed C57BL6/j background (backcrossed for 3 generations only, as further backcrossing onto the C57BL6/j background causes infertility in this genotype) were used for all of the experiments. Mice were housed in groups of 4 per cage in a temperature-controlled room with a 12/12 light/dark cycle. Food and water were provided *ad libitum*.

For the isoproterenol infusion experiments, 3-month-old male wild-type mice from the colony were treated with isoproterenol (Sigma-Aldrich, Mississauga, Canada) at a dose of 60 mg/kg/day or saline for two weeks using the Alzet Osmotic Pumps (Model 2002, Durect Corporation, USA) and maintained in the Animal Care Facility for an additional two weeks. All animals used in these studies were maintained at the University of Western Ontario (UWO, London, Canada) and experiments were performed following the guidelines and protocols approved by the University Council on Animal Care (UCAC) for animal research.

### RNA Microarray Analysis

All sample labelling and GeneChip processing was performed at the London Regional Genomics Centre (Robarts Research Institute, London, Ontario, Canada; http://www.lrgc.ca). RNA quality was assessed using the Agilent 2100 Bioanalyzer (Agilent Technologies Inc., Palo Alto, CA) and the RNA 6000 Nano kit (Caliper Life Sciences, Mountain View, CA). Single stranded complimentary DNA (cDNA) was prepared from 2.0 µg (ventricle extracts) of total RNA as per the Affymetrix GeneChip Whole Transcript (WT) Sense Target Labeling Assay Manual (Affymetrix, Santa Clara, CA). 5.5 µg of single stranded DNA was synthesized, end labeled and hybridized, for 16 hours at 45°C, to Mouse Gene 1.0 ST arrays to probe a set of 33,000 genes. All liquid handling steps were performed by a GeneChip Fluidics Station 450 and GeneChips were scanned with the GeneChip Scanner 3000 7G (Affymetrix, Santa Clara, CA) using Command Console v1.1. Probes were summarized to gene level data in Partek Genomics Suite v6.5 (Partek, St. Louis, MO) using the RMA algorithm [10]. Partek was used to determine gene level ANOVA p-values, fold changes and GO (Gene Ontology) enrichment, using a Fisher’s exact test. Differentially expressed genes were selected based on an ANOVA p-value of less than 0.05 and 1.3 fold increase or decrease between WT and KD samples. The data discussed in this publication have been deposited in NCBI’s Gene Expression Omnibus and are accessible through GEO Series accession number GSE37458 (http://www.ncbi.nlm.nih.gov/geo/query/acc.cgi?acc=GSE37458).

### Quantitative RT-PCR

Total RNA was extracted using the Fatty and Fibrous Tissue RNA Extraction Kit (Bio-Rad Laboratories, Mississauga, Canada) according to the manufacturer’s protocol. Total RNA from whole hearts was eluted in 80 µl of Elution Solution. Quantification and quality analysis of RNA in the extracted samples was done by microfluidic analysis using the Agilent 2100 Bioanalyzer (Agilent Technologies Inc., Palo Alto, CA). All RNA samples used for the reverse transcription reaction had an RNA integrity number of ≥8.0. 20 µl of cDNA was synthesized from 500 ng of total RNA using the High Capacity cDNA Reverse Transcription Kit (Applied Biosystems, Streetsville, Canada) following the manufacturer’s instructions. cDNA was subsequently subjected to qPCR on a CFX-96 Real Time System (Biorad) using the iQ SYBR GREEN SUPERMIX (Biorad). The PCRs were cycled 40 times after initial denaturation (95°C, 3 min) with the following parameters: 95°C for 10 s, annealing and extension at 60°C for 30 s. For each experiment, a non-template reaction was used as a negative control. Relative quantification of gene expression was done with the DD*CT* method using β-actin gene expression to normalize the data. All primers used for qPCR were validated before use to confirm that efficiency was within the required range. Sequences of primers used are available upon request.

### Immunoblotting

Whole hearts were placed in modified ice-cold RIPA buffer and homogenized using a motorized Dounce homogenizer. 40–80 µg of protein lysates were separated using SDS-PAGE and transferred onto PVDF membranes. Antibodies, dilutions and their sources are as follows: anti-acetyl-CoA carboxyase (1∶1000; Cell Signaling), anti-ATP citrate lyase (1∶1000; Cell Signaling), anti-fatty acid synthase (1∶1000; Cell Signaling), anti-purine nucleoside phosphorylase (1∶200; Santa Cruz Biotechnology), anti-α-tubulin (1∶3000; Sigma-Aldrich), and anti-α-actinin (1∶2000; Sigma-Aldrich). α-tubulin or α-actinin was used as a loading control for all samples and the protein was quantified using densitometry analysis.

### Cardiomyocyte Isolation

Adult ventricular myocytes were isolated as previously described [11]. Briefly, hearts were rapidly removed and perfused via the Langendorff method with Ca^2+^-free modified Tyrode solution until the blood was cleared. Hearts were then perfused with Tyrode solution containing 100 µM CaCl_2_ along with 1.4 mg/ml collagenase (type 2; Worthington, Lakewood, NJ) and 0.04 mg/ml protease (type XIV; Sigma, St. Louis, MO) until they were soft (∼10 min). The hearts were removed from the perfusion apparatus, minced into ∼1- mm chunks, and stirred for 4 min in Tyrode solution containing 0.1 mM CaCl_2_, 0.7 mg/ml collagenase, and 0.02 mg/ml protease. Cells were filtered through a 200- µm mesh to remove tissue chunks, and extracellular Ca^2+^ concentration was raised to 0.5 mM over 10 min through three centrifuge cycles.

### ROS Measurements

MitoSOX Red (Invitrogen) was used to measure mitochondrial reactive oxygen species (ROS) production. Isolated cardiomyocytes were loaded with MitoSOX Red (3 µmol/l) in DMEM for 20 min at room temperature, followed by washout. Confocal images were obtained by excitation at 514 nm and measuring the emitted light at 585 nm in cardiomyocytes bathed in normal Tyrode’s solution. The confocal imaging was performed with a Zeiss LSM 510META confocal microscope (CEMEL-Confocal Microscopy Facility, ICB/UFMG).

### Statistical Analyses

Results of qPCR, immunoblotting and MitoSOX experiments are provided as mean ± SEM. The student’s t-test was used to assess statistical differences between two experimental groups using SigmaStat software. P<0.05 was considered statistically significant.

## Results

### Analysis of Transcriptional Alterations in VAChT KD^HOM^ Hearts

We have previously demonstrated that VAChT KD^HOM^ mice with reduced cholinergic neurotransmission develop cardiac dysfunction [5]. To determine the transcriptional alterations in VAChT mutant mice, we performed a microarray analysis using total RNA isolated from ventricles obtained from VAChT KD^HOM^ and age-matched WT mice. Ventricular tissue was used for the microarray analysis due to the fact that the previous experiments with these mice revealed major alterations related to cardiac dysfunction in isolated, ventricular cardiomyocytes [5]. Therefore, we wanted to determine whether there were any changes in gene expression in ventricular tissue that may contribute to this dysfunction. A list of differentially expressed genes was generated by limiting the fold change to at least 1.3 with a significance of p≤0.05 **(Table S1)**. The gene ontology (GO) analysis did not reveal robust alterations in any specific pathway in the mutant ventricles. However, a total of 71 genes showed differential expression between VAChT KD^HOM^ and WT mice, of which 52 genes were significantly up-regulated and 19 down-regulated.

**Figure 1:**
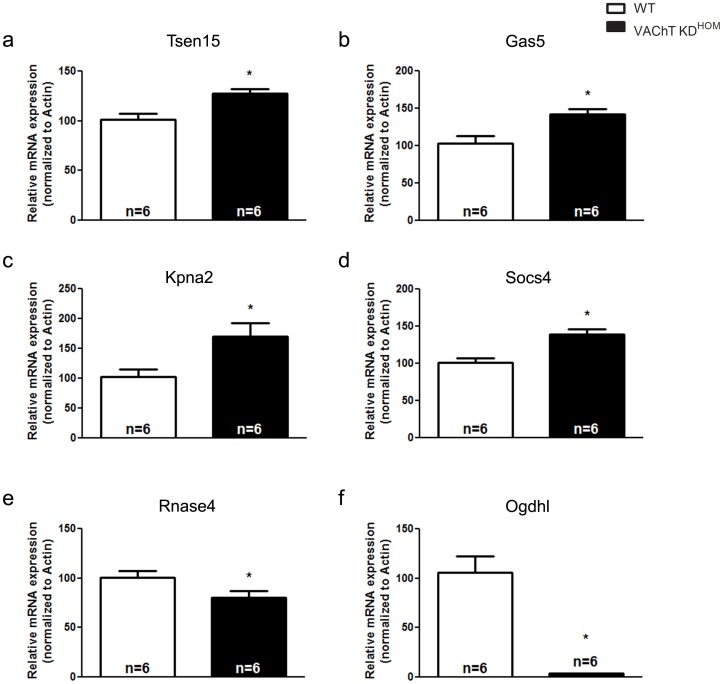
qPCR analysis confirms the expression pattern of several genes detected in the microarray analysis. mRNA expression of tRNA splicing endonuclease 3 (Tsen15; **panel a**), Growth arrest specific 5 (Gas5; **panel b**), karyopherin alpha 2 (Kpna2; **panel c**) and suppressor of cytokine signaling 4 (Socs4; **panel d**) was increased in mutant mice. mRNA analysis of ribonuclease, RNase A family 4 (Rnase4; **panel e**) and oxoglutarate-dehydrogenase like (Ogdhl; **panel f**) confirmed transcriptional downregulation in whole heart RNA from the mutant animals. Data represent the mean ± SEM, with n indicated within bars. *p<0.05 versus wild-type mice.

**Figure 2:**
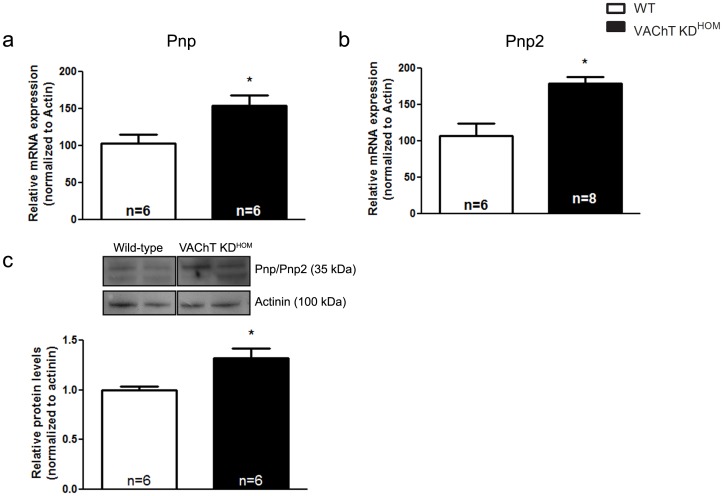
Purine nucleoside phosphorylases are upregulated in the hearts of VAChT KD^HOM^ mice. mRNA expression of purine nucleoside phosphorylase (Pnp; **panel a**) and purine nucleoside phosphorylase 2 (Pnp2, **panel b**) were upregulated. Pnp/Pnp2 protein content appears to upregulated in VAChT KD^HOM^ animals as compared to wild-type mice (**panel c**). Data represent the mean ± SEM, with n indicated within bars. *p<0.05 versus wild-type mice.

To examine the robustness of our microarray results, we performed quantitative real-time PCR (qPCR) on four randomly chosen genes which demonstrated a significant increase (Tsen15, Gas5, Kpna2, Socs4) and two genes which demonstrated a significant decrease (Rnase4, Ogdhl) in the microarray analysis. The qPCR results confirmed that the expression of Tsen15, Gas5, Kpna2 and Socs4 were significantly increased and that of Rnase4 and Ogdhl were significantly decreased in the ventricles obtained from VAChT KD^HOM^ mice **(** **Figure 1** **)**, a result which confirms our microarray data. Further analysis of the ventricular microarray suggested a significant upregulation in the expression of two purine nucleoside phosphorylases, Pnp and Pnp2. Both Pnp and Pnp2 are involved in generating hypoxanthine, a metabolite of adenosine [12]. Hypoxanthine, produced in endothelial cells, has been shown to translocate to myocytes [13], [14] and contribute to production of oxygen free radicals following further metabolism to xanthine and urate [15], [16], [17], [18]. Several studies have previously shown that increased ROS levels can play a major role in cardiac dysfunction in different cardiomyopathies Therefore, due to the potential role of these enzymes in contributing to cardiac malfunction in VAChT KD^HOM^ mice, the increased expression of these enzymes was further confirmed via qPCR **(** **Figure 2a, 2b** **)**. Importantly, this transcriptional upregulation in Pnp/Pnp2 led to a significant increase in the protein content of Pnp/Pnp2 in ventricular tissues obtained from VAChT mutant mice **(** **Figure 2c** **)**.

### Mitochondrial Superoxide Levels are Increased in VAChT KD^HOM^ Animals

**Figure 3:**
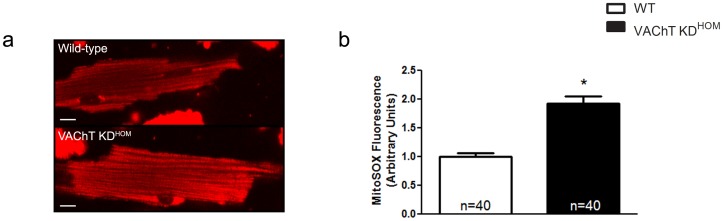
VAChT KD^HOM^ cardiomyocytes show increased levels of ROS. Isolated cardiomyocytes loaded with a MitoSOX superoxide indicator reveal greater ROS levels in mutant myocytes (sample image; **panel a**). A robust, significant increase in fluorescence was observed in the KD cardiomyocytes as compared to wild-type control cells (**panel b**). Data represent the mean ± SEM, with n indicated within bars. *p<0.05 versus wild-type mice. Scale bar = 10 µm.

The increased expression of Pnp/Pnp2 in the hearts of mutant mice might predict a greater production of ROS (as determined via superoxide formation) in these mice. Interestingly, cardiomyocytes from VAChT KD^HOM^ hearts showed a significant increase in superoxide levels as compared to wild-type control cells **(** **Figure 3** **)**, suggesting greater levels of ROS production in VAChT KD cardiomyocytes as compared to WT mice.

### Lipid Biosynthesis Appears to be Unaltered in VAChT KD^HOM^ Mice

**Figure 4:**
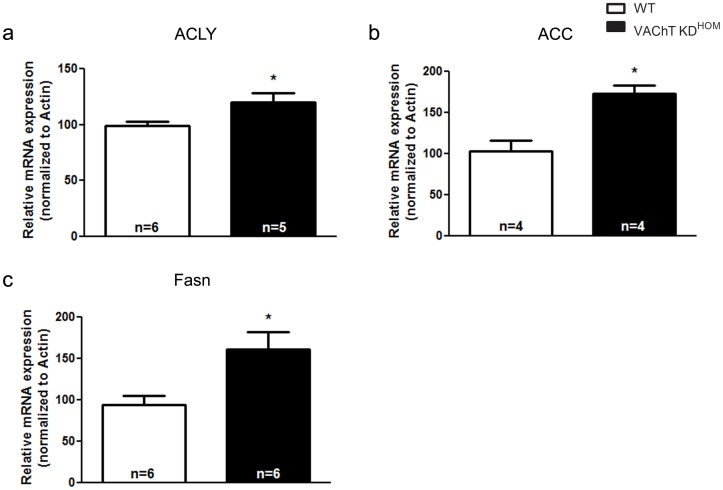
The transcription of genes related to fatty acid biosynthesis is upregulated. mRNA expression of ATP citrate lyase (ACLY, **panel a**), Acetyl-CoA carboxylase (ACC; **panel b**) and fatty acid synthase (FAS; **panel c**) was increased expression in VAChT KD^HOM^ mice. Data represent the mean ± SEM, with n indicated within bars. *p<0.05 versus wild-type mice.

**Figure 5:**
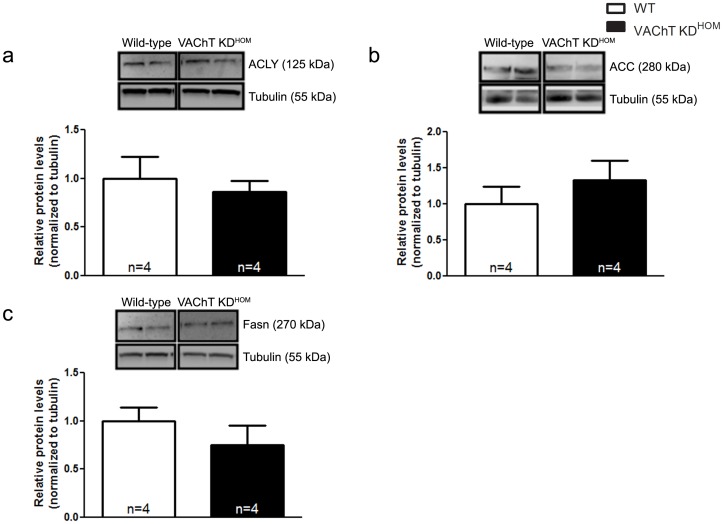
There are no alterations in the protein levels of enzymes involved in lipid biosynthesis. Immunoblotting analysis of ATP citrate lyase (ACLY, **panel a**), Acetyl-CoA carboxylase (ACC; **panel b**) and fatty acid synthase (FAS; **panel c**) revealed no differences in the protein levels of these enzymes in VAChT KD^HOM^ mice. Data represent the mean ± SEM, with n indicated within bars. *p<0.05 versus wild-type mice.

In addition to changes in ROS, which may contribute to phenotype in these mice, previous work has suggested a decrease in fatty acid oxidation during heart failure can also contribute to further decline in cardiac function [19], [20], [21], [22]. In order to examine potential other changes in transcripts that could affect cardiac energetics in the VAChT mutant mice, we used a candidate gene approach and analyzed specific genes involved in the lipid biosynthetic process. In particular, we studied the expression of ATP citrate lyase (ACLY), acetyl-CoA carboxylase (ACC) and fatty acid synthase (FAS). These genes, related to cardiac metabolism, are known to be altered in severe heart failure [23]. ACLY is responsible for converting citrate into oxaloacetate in the cytoplasm and, in the process, generates a molecule of Acetyl-CoA which is utilized by ACC to produce Malonyl-CoA, the substrate used for the generation of long chain fatty acids by FAS. Real-time PCR revealed a significant upregulation in the expression of all three of these genes in VAChT-mutant mice **(** **Figure 4** **)**. However, assessment of protein expression levels using immunoblotting revealed no significant differences in the protein levels of these enzymes between WT and VAChT mutant mice **(** **Figure 5** **)**.

### Analysis of Transcriptional Alterations in Isoproterenol-treated Hearts

**Figure 6:**
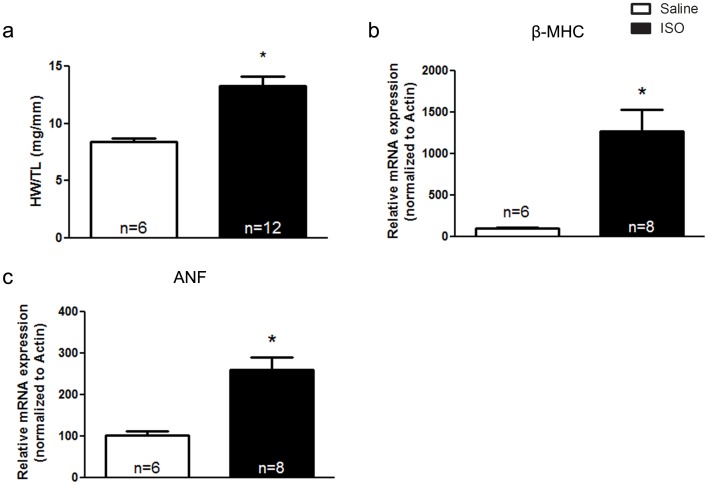
Chronic treatment with isoproterenol (ISO)-induces cardiac remodeling in wild-type mice. Two week ISO treatment led to a significant increase in heart weight/tibia length ratio as compared to saline-treated control mice (**panel a**). The expression of β-myosin heavy chain (β-MHC, **panel b**) and atrial natriuretic factor (ANF, **panel c**) were significantly upregulated in ISO-treated mice. Data represent the mean ± SEM, with n indicated within bars. *p<0.05 versus wild-type mice.

**Figure 7:**
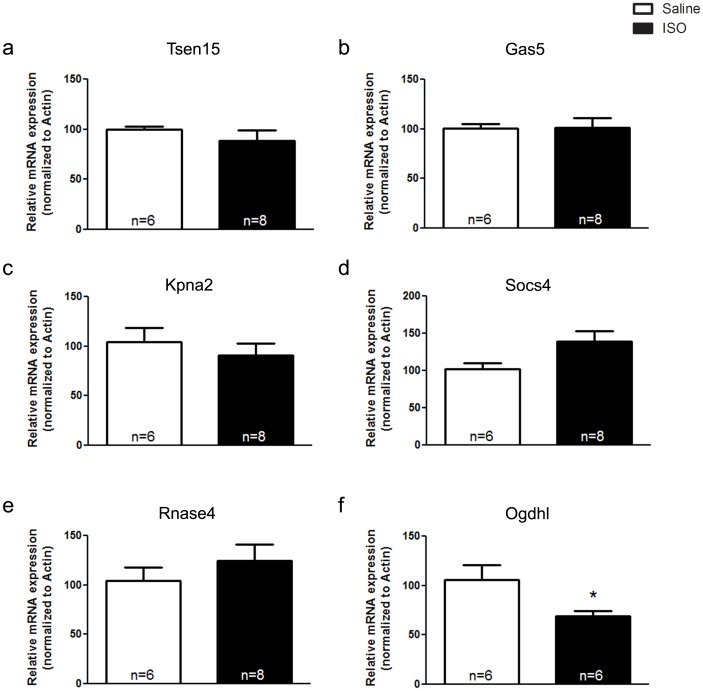
ISO treatment does not lead to the same transcriptional alterations observed in VAChT KD^HOM^ mice. mRNA expression of tRNA splicing endonuclease 3 (Tsen15; **panel a**), Growth arrest specific 5 (Gas5; **panel b**), karyopherin alpha 2 (Kpna2; **panel c**), suppressor of cytokine signaling 4 (Socs4; **panel d**), ribonuclease, RNase A family 4 (Rnase4; **panel e**) and oxoglutarate-dehydrogenase like (Ogdhl; **panel f**) were not significantly altered in the ISO-treated mice as compared to saline-treated controls. Data represent the mean ± SEM, with n indicated within bars. *p<0.05 versus wild-type mice.

**Figure 8:**
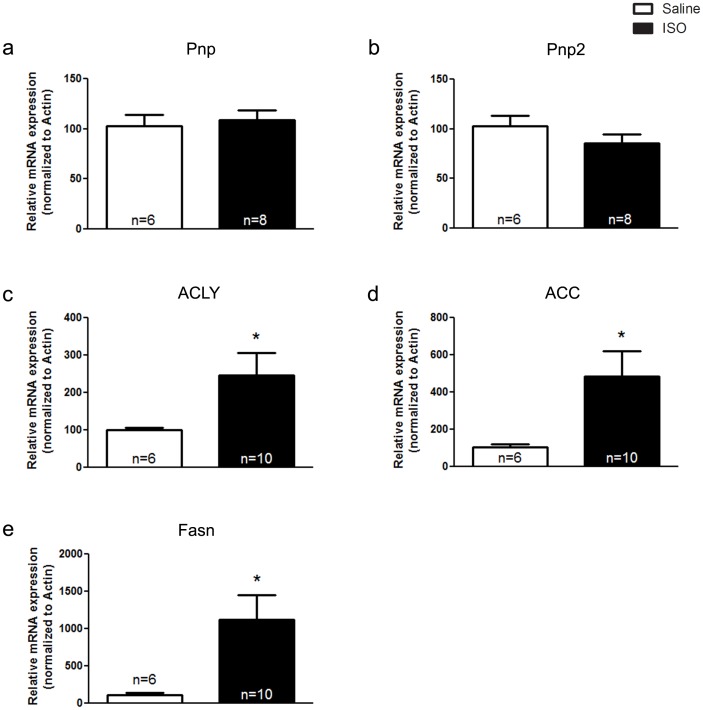
Expression of genes related to fatty acid synthesis is increased following isoproterenol (ISO) treatment. mRNA expression of purine nucleoside phosphrylases (Pnp and Pnp2; **panels a** and **panel b**) were not altered following ISO treatment. ATP citrate lyase (ACLY; **panel c**), acetyl-CoA carboxylase (ACC; **panel d**) and fatty acid synthase (FAS; **panel e**) expression were all significantly increased in ISO-treated mice as compared to saline-treated controls. Data represent the mean ± SEM, with n indicated within bars. *p<0.05 versus wild-type mice.

It is not clear whether autonomic imbalance due to reduced cholinergic tone has similar consequences to imbalance due to increased sympathetic tone. To examine whether the alterations in gene expression observed in the ventricles of VAChT KD^HOM^ mice were observed in a different model of cardiac dysfunction, we chronically treated wild-type mice with isoproterenol to induce cardiac remodeling. Isoproterenol-treated mice demonstrated a significant increase in heart weight to tibia length ratio as compared to saline-treated mice **(** **Figure 6a** **)**. Furthermore, isoproterenol-treated mice demonstrated a significant increase in the expression of two markers of cardiac stress; namely, β-myosin heavy chain (β-MHC: **Figure 6b**) and atrial natriuretic peptide (ANP; **Figure 6c**). Next we examined potential changes in the expression pattern of genes that displayed altered expression in the VAChT KD^HOM^ mice. Interestingly, most of the genes which were altered in VAChT KD^HOM^ mice were not significantly altered in hearts obtained from isoproterenol-treated mice **(** **Figure 7** **)**. However, the expression of Ogdhl **(** **Figure 7f** **)** was significantly decreased following chronic treatment with isoproterenol, similar to the transcriptional alterations observed in the VAChT mutant mice. The expression of PnP and Pnp2 were not significantly different between isoproterenol-treated and saline-treated mice **(** **Figure 8a, 8b** **)**, suggesting that alterations in these genes are specifically related to a decrease in cholinergic tone.

In contrast to the transcriptional changes found in the microarray experiments, the expression of genes related to the lipid biosynthetic pathway did, in fact, appear to be similarly altered in isoproterenol-treated mice and VAChT KD^HOM^ mice **(** **Figure 8c, 8d, 8e** **)**. Furthermore, the relative change in the expression of these genes was much larger in isoproterenol-treated mice than that observed in VAChT KD^HOM^ hearts. Hence, some, but not all transcriptional alterations are reproduced between isoproterenol-treated mice and VAChT KD^HOM^ mice.

## Discussion

Recent reports have implicated the parasympathetic nervous system in the development and progression of heart failure as well as being a potential therapeutic target in heart disease [24]. Vagal stimulation can improve outcome in experimental heart failure [6] and this can be mimicked by treatment with cholinesterase inhibitors [8]. Our previous studies have demonstrated that mice with a systemic decrease in cholinergic tone develop cardiac dysfunction and exhibit many of the characteristics present in cardiac remodeling [5]. Importantly, in VAChT KD^HOM^ mice, these cardiac defects are ameliorated by cholinesterase inhibitor treatment, implicating release of ACh rather than developmentally-induced changes in the control of heart function. Interestingly, in heart failure, transdifferentiation of sympathetic neurons into a cholinergic phenotype has recently been demonstrated, and this appears to have a protective role [9]. However, the role of acetylcholine in controlling long-term cardiac function is still poorly understood.

The present study examined whether the decrease in parasympathetic tone in VAChT KD^HOM^ mice leads to alterations in cardiac gene expression which may contribute to the observed cardiac dysfunction. Our microarray analysis revealed a number of transcriptional changes with a total of 71 genes being significantly different between these mice. Interestingly, transcript and protein levels of two purine nucleoside phosphorylases (Pnp and Pnp2) were significantly increased in the VAChT mutant mice. Both Pnp and Pnp2 are important enzymes responsible for the conversion of inosine to hypoxanthine and have also been shown to metabolize adenosine into adenine, especially under conditions of cardiac stress [12], [25], [26].

The increased levels of Pnp and Pnp2 in VAChT KD^HOM^ mice may lead to the increased production of hypoxanthine in mutant hearts [12]. Interestingly, endothelial cells in the heart appear to be responsible for the majority of adenosine uptake [27]. In addition, they are responsible for the metabolism of adenosine into several compounds, including hypoxanthine [28]. Increased levels of this adenosine metabolite may serve a key role in the cardiac dysfunction observed in mutant mice. For example, hypoxanthine, produced in endothelial cells, can be taken up by the ENT and ENBT1 transporters into myocytes [13], [14], where it can be further metabolized into xanthine and urate [12]. These metabolites contribute to the production of ROS [15], [16], [17], [18], increased levels of which have been shown to play a role in cardiac and vascular dysfunction in both ischemic and non-ischemic cardiomyopathies [16], [18], [29], [30]. Interestingly, we observed an increase in the levels of ROS in ventricular cardiomyocytes isolated from VAChT KD^HOM^ mice. Oxygen free radicals contribute to declining cardiac function during heart failure via many different mechanisms and result in damage to the myocardium [31], [32]. They can also have detrimental effects specifically in cardiomyocytes as they can activate cell death through both necrotic and apoptotic pathways [33], [34]. Future studies will be necessary to further characterize the mechanisms which lead to increased ROS production in the mutant mice as well as determine the physiological importance of these oxygen free radicals and their role in the observed cardiac dysfunction. However, it is tempting to speculate that these alterations may play a role in the dysfunction found in VAChT mutant mice.

It is important to note that ADP levels are upregulated in heart failure suggesting that failing cardiac tissue utilizes greater amounts of ATP [35]. Under normal conditions, the vasodilatory actions of adenosine may be able to compensate for this increased utilization of ATP by myocardial tissue. However, VAChT KD^HOM^ mice show increased levels of Pnp which has previously been shown to metabolize adenosine into adenine [25], [26]. This may contribute to the inability of the mutant hearts to maintain normal contractile function; an idea which is in accordance with previous research suggesting that the failing heart is energy-starved [36], [37]. It should be noted that these alterations seems to be selectively related to decreased cholinergic function, as the changes observed in the microarray experiments were not observed in isoproterenol-treated mice.

Significant alterations in substrate metabolism have been observed during the progression of heart failure and it is suggested that these changes contribute to cardiac remodeling and dysfunction observed during disease progression [38]. Previous studies have shown that, in end stage heart failure, there is an increase in glucose oxidation coupled with a decrease in fatty acid oxidation and these changes in substrate utilization lead to adverse effects during late stage heart failure [19], [20], [21], [22].

To further examine transcriptional alterations in VAChT mutant hearts that may not have been identified in the microarray, we chose genes related to the lipid biosynthetic pathway (ACLY, ACC and FAS). These pathways have been previously found to be altered in heart failure [23]. In agreement with the notion in cardiac dysfunction these pathways may be altered, chronic treatment with the β-agonist isoproterenol, which mimics the sympathetic overactivation observed in several cardiac diseases, increased mRNA levels for ACLY, ACC and FAS several fold. We also found an increase in mRNA expression of ACLY, ACC and FAS in VAChT mutant mice, suggesting at least some similarities between autonomic imbalance due to decreased cholinergic tone and sympathetic overactivation. Although gene expression changes were confirmed for several genes involved in the generation of long-chain fatty acids in VAChT-mutant mice, the protein levels for these enzymes appeared to be unaltered, although we cannot discard the possibility that their turnover might be increased.

It is important to note that VAChT KD^HOM^ animals exhibit a global decrease in VAChT levels and, therefore, decreased cholinergic tone. This is significant because it has recently been proposed that cardiomyocytes possess the machinery (VAChT, ChAT and CHT1) for *de novo* production of ACh [39], [40] and are able to synthesize and release this neurotransmitter. This non-neuronal ACh may then act in an autocrine/paracrine fashion to amplify neuronal cholinergic signaling [40]. We have recently demonstrated that this non-neuronal cardiomyocyte release of ACh plays an important role in the protection of myocytes against isoproterenol-induced hypertrophy and that VAChT mutant mice are deficient in non-neuronal ACh secretion as well [41]. We cannot discard the possibility that the gene alterations we uncovered here may, at least in part, be due to deficient ACh release from cardiomyocytes. Future studies will be necessary to specifically analyze the importance of this non-neuronal cholinergic system in myocytes and its contribution to the cardiac dysfunction observed after reduced cholinergic tone. This may provide an unanticipated mechanism by which non-neuronal ACh can play an important role in cardiac function.

## Supporting Information

Table S1 **Genes which show transcriptional alterations in ventricles from VAChT KD^HOM^ mutant mice.**
(PDF)
