# Supplementary material for: An Analysis of the Myocardial Transcriptome in a Mouse Model of Cardiac Dysfunction with Decreased Cholinergic Neurotransmission
Source: PLoS One. 2012 Jun 29;7(6):e39997. doi: 10.1371/journal.pone.0039997 (PMC3386908; doi:10.1371/journal.pone.0039997)
Supplement: Table S1 — Genes which show transcriptional alterations in ventricles from VAChT KDHOM mutant mice. (PDF) [file pone.0039997.s001.pdf]

Table S1 – Genes which show transcriptional alterations in ventricles from VAChT KD^{HOM} mutant mice.

Gene Name	Gene Symbol	Transcript ID	RefSeq ID	Normalized fold-change
**tRNA splicing endonuclease 15 homolog (S. cerevisiae)**	Tsen15	10536593	NM_025677	1.89193
**cyclin B1 interacting protein 1**	Ccnb1ip1	10419465	NM_001111119	1.84595
**purine-nucleoside phosphorylase**	Pnp	10414514	NM_013632	1.73353
**growth arrest specific 5**	Gas5	10351026	NR_002840	1.58404
**karyopherin (importin) alpha 2**	Kpna2	10497503	NM_010655	1.56091
**small nucleolar RNA, C/D box 1C**	Snord1c	10382844	NR_028569	1.55143
**purine-nucleoside phosphorylase 2**	Pnp2	10414527	NM_001123371	1.5345
**MU-2/AP1M2 domain containing, death-inducing**	Mudeng	10414449	NM_144535	1.53211
**ribonuclease, RNase A family, 1 (pancreatic)**	Rnase1	10419563	NM_011271	1.46911
**mitochondrial ribosomal protein L47**	Mrpl47	10497703	NM_029017	1.43058
**membrane-spanning 4-domains, subfamily A, member 6D**	Ms4a6d	10466210	NM_026835	1.41185
**calmodulin binding transcription activator 1**	Camta1	10518835	NM_001081557	1.40688
**small nucleolar RNA, C/D box 34**	Snord34	10563110	NR_002455	1.40542
**acyl-CoA thioesterase 10**	Acot10	10427883	NM_022816	1.40355
**LysM, putative peptidoglycan-binding, domain containing 2**	Lysmd2	10587226	NM_027309	1.40225
**suppressor of cytokine signaling 4**	Socs4	10414350	NM_080843	1.39699
**centrosome and spindle pole associated protein 1**	Cspp1	10344817	NM_026493	1.39383
**listerin E3 ubiquitin protein ligase 1**	Ltn1	10440568	NM_001081068	1.39239
**killer cell lectin-like receptor subfamily A, member 9**	Klra9	10548511	ENSMUST00000088019	1.39002
**protein tyrosine phosphatase-like A domain containing 1**	Ptplad1	10594501	NM_021345	1.38888
**profilin 2**	Pfn2	10498309	NM_019410	1.38519
**LLP homolog, long-term synaptic facilitation (Aplysia)**	Llph	10515293	NM_025431	1.3812
**triadin**	Trdn	10362454	NM_029726	1.37467
**COBW domain containing 1**	Cbwd1	10466818	NR_033744	1.37322
**S-adenosylmethionine decarboxylase 1**	Amd1	10447510	NM_009665	1.3687
**LLP homolog, long-term synaptic facilitation (Aplysia)**	Llph	10603702	NM_025431	1.36867
**mitochondrial ribosomal protein L20**	Mrpl20	10511149	NM_025570	1.3685
**phosphatidylserine decarboxylase, pseudogene 2**	Pisd-ps2	10447517	NR_003519	1.36647
**microRNA 208a**	Mir208a	10419932	NR_029724	1.35736
**NADH dehydrogenase (ubiquinone) 1 alpha subcomplex**	Ndufaf1	10486284	NM_027175	1.35388
**membrane-spanning 4-domains, subfamily A, member 6B**	Ms4a6b	10461622	NM_027209	1.34442

**Mki67 (FHA domain) interacting nucleolar phosphoprotein**	Mki67ip	10349239	NM_026472	1.34429
**La ribonucleoprotein domain family, member 7**	Larp7	10514383	NM_138593	1.3437
**ribonuclease H2, subunit B**	Rnaseh2b	10415791	NM_026001	1.34199
**tubulin cofactor A**	Tbca	10406710	NM_009321	1.337
**FAST kinase domains 1**	Fastkd1	10483521	NM_177244	1.32967
**chromatin modifying protein 5**	Chmp5	10504008	NM_029814	1.32433
**mitochondrial ribosomal protein L22**	Mrpl22	10376320	NM_175001	1.32406
**SH3-binding domain glutamic acid-rich protein**	Sh3bgr	10437180	NM_015825	1.32303
**DCN1, defective in cullin neddylation 1, domain containing 5**	Dcun1d5	10583034	NM_029775	1.31991
**M phase phosphoprotein 6**	Mphosph6	10478283	NM_026758	1.31718
**DnaJ (Hsp40) homolog, subfamily B, member 11**	Dnajb11	10434675	NM_001190804	1.3166
**HIG1 domain family, member 1A**	Higd1a	10597871	NM_019814	1.31585
**polymerase (DNA directed), iota**	Poli	10459655	NM_011972	1.31454
**small nucleolar RNA, H/ACA box 26**	Snora26	10522465	NR_031758	1.31066
**mediator of RNA polymerase II transcription, subunit 31 homolog**	Med31	10388154	NM_026068	1.31001
**cAMP-regulated phosphoprotein 19**	Arpp19	10587104	NM_021548	1.30743
**TDP-glucose 4,6-dehydratase**	Tgds	10422259	NM_029578	1.30694
**FtsJ methyltransferase domain containing 1**	Ftsjd1	10575376	NM_146215	1.30622
**isopentenyl-diphosphate delta isomerase**	Idi1	10482762	NM_145360	1.30457
**transmembrane protein 19**	Tmem19	10372488	NM_133683	1.3031
**E2F transcription factor 6**	E2f6	10394690	NM_033270	1.30278
**olfactory receptor 1087**	Olfr1087	10484626	NM_146846	-1.30118
**vomeronasal 1 receptor 210**	Vmn1r210	10408150	NM_134235	-1.30189
**protease, serine-like 1**	Prssl1	10370644	NM_001042710	-1.30456
**microRNA 501**	Mir501	10603304	NR_030496	-1.30937
**ninein**	Nin	10396146	NM_008697	-1.31064
**LIM homeobox protein 2**	Lhx2	10471833	NM_010710	-1.31587
**zinc finger protein 160**	Zfp160	10442177	ENSMUST00000088811	-1.31771
**thymocyte selection associated**	Themis	10362350	NM_178666	-1.3193
**olfactory receptor 229**	Olfr229	10584516	NM_146613	-1.31993
**angiopoietin-like 4**	Angptl4	10450038	NM_020581	-1.32425
**taste receptor, type 2, member 115**	Tas2r115	10548665	NM_207020	-1.32888
**3-phosphoglycerate dehydrogenase**	Phgdh	10500529	NM_016966	-1.32932
**transmembrane protein 121**	Tmem121	10399021	NM_153776	-1.34042

**neuropilin (NRP) and tolloid (TLL)-like 1**	Neto1	10457091	NM_144946	-1.34581
**cyclin M2**	Cnnm2	10468249	NM_033569	-1.3752
**peripherin 2**	Prph2	10445633	NM_008938	-1.38184
**ribonuclease, RNase A family 4**	Rnase4	10414537	NM_021472	-1.4077
**late cornified envelope 1C**	Lce1c	10493889	NM_028622	-1.42251
**oxoglutarate dehydrogenase-like**	Ogdhl	10413874	NM_001081130	-7.53128
